# *Lawsonia intracellularis* exploits β-catenin/Wnt and Notch signalling pathways during infection of intestinal crypt to alter cell homeostasis and promote cell proliferation

**DOI:** 10.1371/journal.pone.0173782

**Published:** 2017-03-21

**Authors:** Yang W. Huan, Rebecca J. Bengtsson, Neil MacIntyre, Jack Guthrie, Heather Finlayson, Sionagh H. Smith, Alan L. Archibald, Tahar Ait-Ali

**Affiliations:** 1 The Roslin Institute, University of Edinburgh, Easter Bush Campus, Roslin, Midlothian, United Kingdom; 2 Royal (Dick) School of Veterinary Studies, University of Edinburgh, Easter Bush Campus, Roslin, Midlothian, United Kingdom; University of Kansas School of Medicine, UNITED STATES

## Abstract

*Lawsonia intracellularis* is an obligate intracellular bacterial pathogen that causes proliferative enteropathy (PE) in pigs. *L*. *intracellularis* infection causes extensive intestinal crypt cell proliferation and inhibits secretory and absorptive cell differentiation. However, the affected host upstream cellular pathways leading to PE are still unknown. β-catenin/Wnt signalling is essential in maintaining intestinal stem cell (ISC) proliferation and self-renewal capacity, while Notch signalling governs differentiation of secretory and absorptive lineage specification. Therefore, in this report we used immunofluorescence (IF) and quantitative reverse transcriptase PCR (RTqPCR) to examine β-catenin/Wnt and Notch-1 signalling levels in uninfected and *L*. *intracellularis* infected pig ileums at 3, 7, 14, 21 and 28 days post challenge (dpc). We found that while the significant increase in Ki67^+^ nuclei in crypts at the peak of *L*. *intracellularis* infection suggested enhanced cell proliferation, the expression of *c-MYC* and *ASCL2*, promoters of cell growth and ISC proliferation respectively, was down-regulated. Peak infection also coincided with enhanced cytosolic and membrane-associated β-catenin staining and induction of *AXIN2* and *SOX9* transcripts, both encoding negative regulators of β-catenin/Wnt signalling and suggesting a potential alteration to β-catenin/Wnt signalling levels, with differential regulation of the expression of its target genes. We found that induction of *HES1* and *OLFM4* and the down-regulation of *ATOH1* transcript levels was consistent with the increased Notch-1 signalling in crypts at the peak of infection. Interestingly, the significant down-regulation of *ATOH1* transcript levels coincided with the depletion of *MUC2* expression at 14 dpc, consistent with the role of *ATOH1* in promoting goblet cell maturation. The lack of significant change to *LGR5* transcript levels at the peak of infection suggested that the crypt hyperplasia was not due to the expansion of ISC population. Overall, simultaneous induction of Notch-1 signalling and the attenuation of β-catenin/Wnt pathway appear to be associated with the inhibition of goblet cell maturation and enhanced crypt cell proliferation at the peak of *L*. *intracellularis* infection. Moreover, the apparent differential regulation of apoptosis between crypt and lumen cells together with the strong induction of Notch-1 signalling and the enhanced *SOX9* expression along crypts 14 dpc suggest an expansion of actively dividing transit amplifying and/or absorptive progenitor cells and provide a potential basis for understanding the development and maintenance of PE.

## Introduction

*Lawsonia intracellularis* is a Gram negative, obligate intracellular bacterial pathogen which infects porcine intestinal crypt cells and causes proliferative enteropathy (PE), an economically significant disease of the pig industry worldwide [1–3]. *L*. *intracellularis* invades the immature intestinal crypt cells where its presence is associated with extensive cell proliferation, disruption to the intestinal mucosal integrity and mucosal thickening [3–8]. Previous studies have shown that hyperplastic crypts are mainly found in the distal small intestine (ileum) at the peak of infection, indicating that this segment of the intestinal tract is the preferential site of infection [2, 6, 9]. There are two main clinical manifestations; acute cases are associated with haemorrhagic diarrhoea and sudden death whereas chronic infection, more common in younger pigs, is typified by wasting and loss of condition that may be accompanied by non-haemorrhagic diarrhoea. In most cases chronic PE is transient [1–3, 6, 8].

A previous study of changes in host gene expression in response to infection using RNA-seq analysis showed that genes promoting active cell division and the immature progenitor/stem cell marker, SOX9, are induced while solute carriers and transporters of matured absorptive enterocytes are down-regulated in *L*. *intracellularis* infected crypt cells [10]. Similarly, analysis of gene expression of *L*. *intracellularis*-infected pig ileal tissues using microarray technology suggested the inhibition of secretory goblet cell and absorptive enterocyte differentiation as well as the induction of cell proliferation at the peak of infection [8]. Moreover, depletion of MUC2 expression observed at the peak of *L*. *intracellularis* infection is associated with the loss of matured goblet cells [4]. Loss of secretory goblet cells and absorptive enterocytes causes disruption to the mucosal integrity and reduced uptake of nutrients, water and ions respectively, which is consistent with the clinical signs of PE [1–8]. However, the affected upstream cellular pathway leading to enhanced crypt cell proliferation, altered cell differentiation and homeostasis is still unknown.

Intestinal epithelium undergoes complete cellular replacement every four to five days [11–12]. The crypts of Lieberkühn house the fast-cycling, LGR5-expressing (LGR5^+^) intestinal stem cells (ISCs) which divide once every one to two days, giving rise to rapidly dividing, transit amplifying (TA) progenitor cells [11–14]. The TA cells then give rise to all the differentiated intestinal cell types categorised into secretory cells (Goblet cells, Tuft cells, enteroendocrine cells, Paneth cells) and absorptive enterocytes [11–14]. The functionality and the architecture of intestinal epithelium depend on the tight balance between cell division and apoptotic cell death, as well as the highly regulated intestinal cell differentiation events [11–14]. These cellular processes are highly regulated by multiple signalling pathways, particularly β-catenin/Wnt and Notch signalling, to maintain the overall intestinal epithelium homeostasis [11–14].

Canonical β-catenin/Wnt signalling is activated by the binding of membrane-bound Wnt ligands to Frizzled receptors which then promotes β-catenin stabilisation and its nuclear translocation [15]. β-catenin then interacts with its binding partners of Transcription Factor/ Lymphoid enhancer-binding factor (TCF/Lef), in the nucleus and regulates target gene expression [15]. High levels of β-catenin/Wnt signalling at the crypt base is essential to maintain proliferation and the self-renewal capacity of ISCs [16–19]. Canonical Notch signalling is activated by the binding of membrane-bound Jagged or Delta-like ligands to Notch 1–4 receptors, which promotes cleavage of the Notch receptor intracellular domain (NICD) by γ-secretase [12]. NICD then translocates into the nucleus and interacts with RPBJ-k and other cofactors regulating its target gene expression [12]. Activated Notch signalling drives the commitment of intestinal TA progenitor cells into the absorptive lineage by suppressing secretory lineage specification through the inhibition of atonal homolog 1 (ATOH1) expression [18–24]. TA progenitors with inactivated Notch signalling will express ATOH1 which drives cell cycle exit and forms secretory progenitor cells [18–21, 23–24]. Moreover, activated Notch signalling is essential in maintaining intestinal stem cell survival and its proliferation in the base of intestinal crypts [19–20, 23]. Constitutive activation of β-catenin/Wnt signalling causes intestinal crypt hyperplasia as seen in loss-of-function mutations of APC gene in various cases of colorectal cancer while ATOH-1 gene knockouts in mice lead to secretory cell dysplasia, which are reminiscent of PE lesions [18, 20–21, 23–25].

The aim of this work was to examine the alterations in goblet cell differentiation and enhanced crypt cell proliferation observed at the peak of *L*. *intracellularis* infection in the context of Notch and β-catenin/Wnt pathways, by using immunofluorescence (IF) and quantitative reverse transcriptase PCR (RTqPCR). IF and RTqPCR were carried out to examine the population of matured goblet cells (by assessing MUC2 expression), crypt cell proliferation and apoptotic cell death (using IF detection of Ki67 and cleaved Caspase-3 respectively) and the activation states of Notch and β-catenin/Wnt pathways in uninfected and infected pig ileums. We report that the simultaneous induction of Notch-1 signalling and alterations to β-catenin/Wnt pathway are associated with the inhibition of goblet cell maturation and enhanced crypt cell proliferation at the peak of infection. This could represent a potential modus operandi for *L*. *intracellularis* invasion and establishment of infection and provide an important basis for understanding the development and maintenance of PE.

## Materials and methods

### Pigs

Pig Ileum samples were from a previous challenge study by Macintyre et al., (2003). Uninfected pigs (both sexes equally represented) aged 7 months old were randomly selected from minimal disease herd and tested negative for *Salmonella spp*, *Yersinia spp*, *Brachyspira hyodysenteriae*, *B*.*pilosicoli* and *L*. *intracellularis* in faecal samples. Pigs were orally challenged by pure *L*. *intracellularis* culture (isolate LR189/5/83). 2–4 pigs were euthanized at 3, 7, 14, 21, 28 days post challenge (dpc). Monoclonal anti-*L*. *intracellularis* antibody (VPM53) was used for IF to stain *L*. *intracellularis* bacteria. Ileum samples from three uninfected, age-matched pigs from a separate herd were used as negative controls.

### Immunofluorescence (IF)

Sections of formalin-fixed ileum from three uninfected controls and two infected pigs per time point at 3, 7, 14 and 28 dpc [8] were used for immunofluorescence. All the antibodies tested (Table 1) except for anti-*L*. *intracellularis* (LI) antibody required heat-mediated antigen retrieval in citrate buffer at pH6, using a Histo5 Rapid Microwave Histoprocessor (Milestone) with the following protocol: isotope retrieval, high pressure and 110°C 5 minutes 20 slides. For LI immunostaining, sections were incubated in proteinase K (DAKO UK Ltd., Ely, UK) for 10 minutes at room temperature, followed by two washes in PBS. 0.10% Triton-X in phosphate buffer saline (PBS) and 0.010% Triton-X in PBS were used to permeabilise the tissue for anti-Sox9, anti-β-catenin, anti-Ki67 and anti-NICD1 staining respectively for 10 minutes at room temperature. Slides were incubated in 5% bovine serum albumin (BSA) in PBS at room temperature for 30 minutes. Excess blocking solution was removed and slides were incubated overnight at 4°C with primary antibodies diluted in 1% BSA in PBS at the concentration stated in Table 1. Negative controls were subjected to the same procedures but with no primary antibody. Slides were washed twice in PBS before one hour incubation at room temperature with secondary antibodies Alexa-Fluor-647 goat anti-rabbit (1:1200, Thermo Fisher Scientific) and Fluorescein isothiocyanate (FITC)-conjugated goat anti-mouse Immunoglobulin G (IgG) (Fc specific) F(ab)2 fragment (1:1500, Sigma-Aldrich) diluted in 1% BSA in PBS. Slides were washed twice in PBS then incubated with 6-diamidino-2-phenylindone (DAPI, 1:500 in water, Thermo Fisher Scientific) at room temperature for 10 minutes. Slides were washed twice in PBS and mounted with Lab Vision PermaFluor aqueous mounting medium (Thermo Fisher Scientific). Sections were observed using LSM700 confocal laser scanning microscope (Carl Zeiss).

**Table 1:** List of antibodies tested.

Antibodies	Source of antibody (Host)	Provider	Dilution	Remarks
*L*. *intracellularis* (LI)	Mouse	University of Edinburgh	1:400	Targeting cell surface transporter of *L*. *intracellularis*
MUC2	Rabbit	Abcam	1:1000	Secretory Mucin (goblet cell marker)
β-catenin	Rabbit	Abcam	1:100	Stains cell-cell junctions, nuclear translocation as proliferation marker
Notch-1 cytoplasmic/intracellular domain (NICD1)	Rabbit	Abcam	1:100	Regulating intestinal epithelial differentiation
Sox9	Rabbit	Millipore	1:1000	Progenitors/Intestinal stem cells marker
Ki67	Mouse	DAKO UK Ltd	1:100	Proliferating cell
Cleaved caspase-3	Rabbit	Cell signalling	1:400	Apoptotic cells

Images were captured and processed using Zen Blue software (Carl Zeiss).

### Fluorescence intensity quantification

ImageJ1.49S (ImageJ, U. S. National Institutes of Health) was used to quantify the fluorescence intensity of LI, MUC2, SOX9 and NICD1 staining. For each time point, the fluorescence intensity of LI and MUC2 staining was measured in 3 random fields (3–4 longitudinal crypts per field). For quantification of SOX9 and NICD1 staining intensity along the upper and lower halves of uninfected and infected crypts at 14 dpc, 15 longitudinal crypts were selected from at least 3 random fields at 63x magnification. Crypts were separated into lower and upper halves of equal length. Integrated density and mean fluorescence were acquired. For each integrated density, an area without fluorescence was selected for background intensity reading. Corrected Total Fluorescence was calculated using the formula, Integrated Density—(Area of selected cell*Mean fluorescence of background readings) [26]. For quantification of KI67^+^- nuclei, 8 longitudinal crypts from 3 randomly selected fields at 63x magnification were selected for uninfected and infected crypts at each time points. ImageJ 1.49S cell counter function was used to count the number of Ki67^+^ nuclei along the crypts.

### RNA samples

RNA samples originated from previous microarray analysis [8]. Total RNA was extracted from ileum samples using Trizol (Invitrogen) following a standard protocol [27]. Qiagen’s RNeasy mini-kit was used to further purify the RNA. The quantity and quality of RNA samples were assessed using a Nanodrop ND1000 spectrophotometer (NanoDrop Technologies Inc., Wilmington, DE, USA) and Agilent 2100 bioanalyser (Agilent Technologies) respectively.

### Real Time quantitative PCR (RTqPCR)

RTqPCR was carried out using RNA samples from uninfected ileums and infected ileums from pigs euthanized at 7, 14, 21 and 28 dpc. Primers (S1 Table) were designed using OligoPerfect^™^ Designer (Thermo Fisher Scientific) and were positioned on exons flanking an intron to reduce the amplification of contaminating genomic DNA. Brilliant III Ultra-Fast SYBR Green RTqPCR Master Mix kit (Agilent Technologies) was used with manufacturer’s protocol. RTqPCR was performed on a Stratagene MX3000P (Stratagene) with thermal profile: 10 minutes at 50°C; 3 minutes at 95°C and 40 cycles of 95°C 20s, 60°C 20s. Each RNA sample was run in duplicate. All RTqPCR results were normalised to GAPDH housekeeping transcript and to uninfected samples [8].

### Statistical analysis

Graphs were generated on Excel (Microsoft, Redmond, WA, USA). Student’s t-test (unequal variance, 2 tailed) was carried out to determine statistical significance. Data are expressed as the means of standard error, ±SEM. Experimental readings (i.e. Fluorescence intensity and mRNA transcript levels) were compared to those of uninfected samples, unless as stated otherwise. p<0.05 is considered as statistically significant. Stars (*) on graphs represents p-values for statistically significant comparisons, with * denotes p<0.05;** denotes p<0.01; * denotes p<0.001; **** denotes p<0.0001.

## Results

### Peak of *L*. *intracellularis* infection

**Fig 1:**
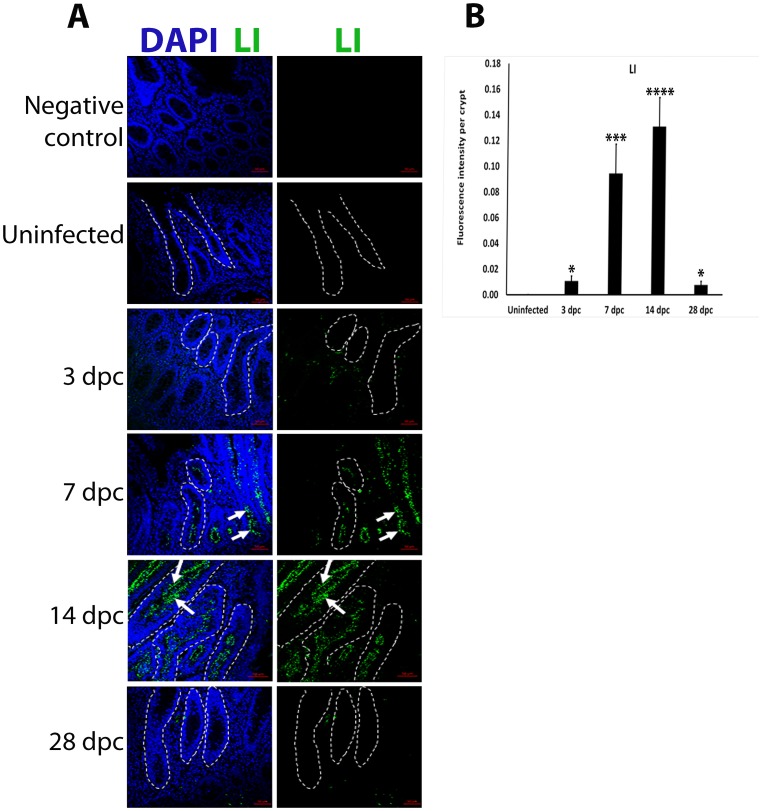
*L*. *intracellularis* bacterial load peaks at 14 dpc. **A)** IF detection of *L*. *intracellularis* bacteria using monoclonal VPM53 antibody in uninfected and *L*. *intracellularis* infected ileums in pigs at 3, 7, 14 and 28 dpc. The presence of anti *L*. *intracellularis* antibodies (VPM53) was detected using FITC-conjugated secondary antibodies (green). Nuclei counterstained with DAPI (Blue). *Left panel*: Merged DAPI (blue) and *L*. *intracellularis*, LI (green) channels, *Right Panel*: LI (green) channel. Some of the crypts are outlined in white dashed lines. White arrows indicate LI labelling along the apical cytosol of crypts cells at 7 and 14 dpc. Scale bar: 50μm. **B)** Quantification of LI staining intensity in uninfected and infected pig ileum crypts at 3,7,14 and 28 dpc. Mean values ± standard error are shown. Y-axis represents fluorescence intensity of LI antigen staining per crypt.

*L*. *intracellularis* infection were evaluated in uninfected and infected pig ileums using IF. There was limited *L*. *intracellularis* staining in crypts at 3 and 28 dpc (Fig 1A). *L*. *intracellularis* bacteria was mostly observed along the whole length of the crypts at 7 and 14 dpc, primarily in the apical cytosol of lining epithelial cells (Fig 1A, white arrows). Fluorescence signal intensity quantification of *L*. *intracellularis* staining confirmed that crypts at 14 dpc harboured the highest pathogen load when compared to uninfected and infected crypts at 3 and 28 dpc (Fig 1B). This indicates 14 dpc as the peak of infection as was observed in previous studies [4, 8].

### Depletion of MUC2 at the peak of *L*. *intracellularis* infection

**Fig 2:**
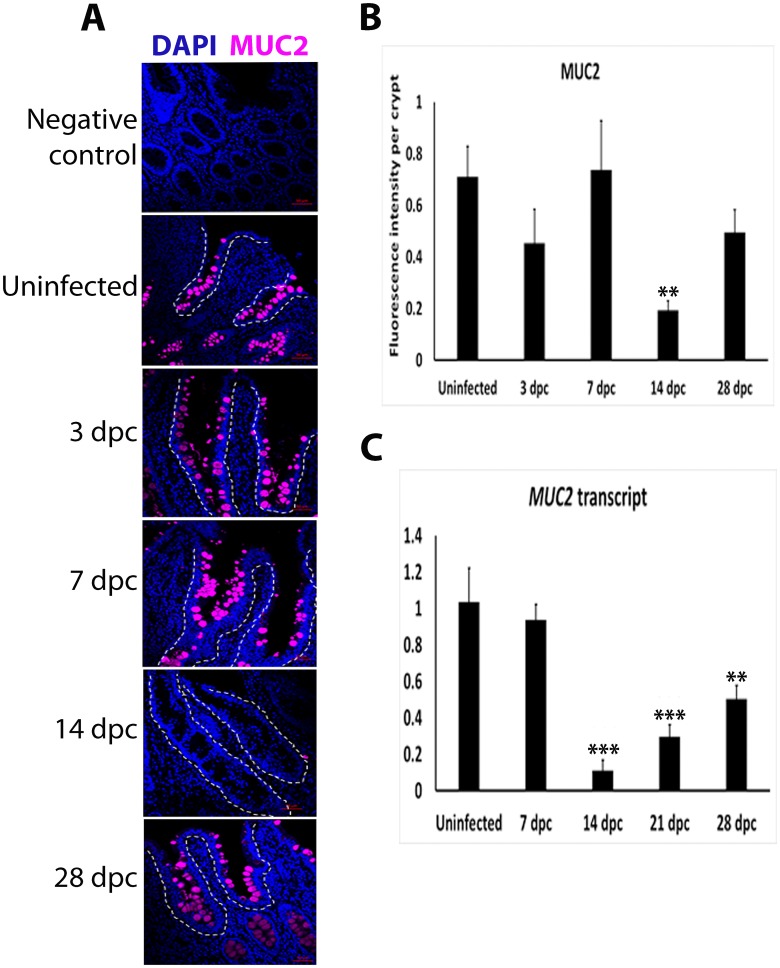
Loss of MUC2 fluorescence signal and expression in *L*. *intracellularis* infected ileum crypts at 14 dpc. **A)** IF detection of MUC2 in uninfected and *L*. *intracellularis* infected pig ileums at 3, 7, 14 and 28 dpc with confocal microscopy. Anti-MUC2 antibody was detected using Alexa-647-conjugated secondary antibody (purple). Nuclei were counterstained by DAPI (blue). Some crypts are outlined in white dashed lines. Scale bar: 50μm. **B)** Quantification of MUC2 fluorescence intensity in uninfected and *L*. *intracellularis* infected crypts at 3, 7, 14 and 28 dpc using ImageJ 1.49S. Mean values ± standard error are shown. Y-axis represents fluorescence intensity of MUC2 antigen staining per crypt. **C)** Level of *MUC2* transcripts in uninfected and *L*. *intracellularis* infected crypts at 7, 14, 21 and 28 dpc. Mean values ± standard error are shown. All RTqPCR results were normalised to GAPDH housekeeping transcript and to uninfected samples [8]. Y-axis represents normalised *MUC2* transcript levels.

Using IF the presence of MUC2 was observed as bright foci representing mucin-containing vacuoles in goblet cells of infected crypts at 3 and 7 dpc and in uninfected crypts (Fig 2A). Bright foci were significantly absent in 14 dpc crypts (Fig 2A and 2B). MUC2 positive foci were observed in lining crypts at 28 dpc, with no significant differences in fluorescence intensity when compared to uninfected crypts (p<0.17). Similarly, *MUC2* transcript levels were significantly down-regulated at 14 dpc compared to other time points and uninfected crypts (Fig 2C). These results support a significant depletion in MUC2 expression at 14 dpc, the peak of infection.

### Increased cell proliferation and apoptosis at the peak of *L*. *intracellularis* infection

**Fig 3:**
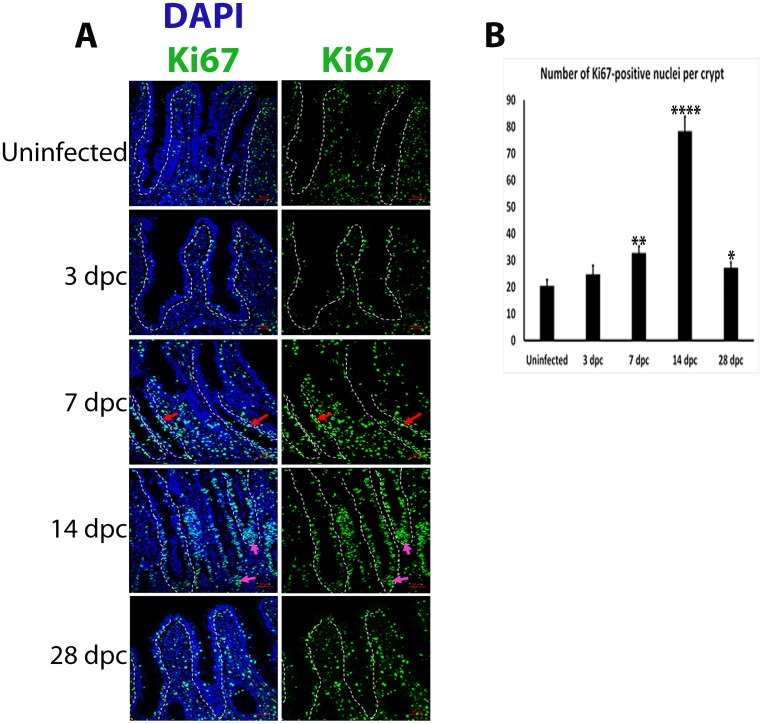
Increase in Ki67+ nuclei in *L*. *intracellularis* infected crypts at 7 d pc and 14 dpc. **A)** IF detection of Ki67 in uninfected and *L*. *intracellularis* infected pig ileums at 3, 7, 14 and 28 dpc using confocal microscopy. Anti-Ki67 antibody was detected using FITC-conjugated secondary antibody (green) and nuclei were counterstained with DAPI (blue). *Left panel*: merged DAPI (blue) and Ki67 (green) channels; *Right panel*: Ki67 channel (green). Some of the crypts are outlined in white dashed lines. White arrows indicate clusters of Ki67^+^ nuclei observed at the mid-regions of *L*. *intracellularis* infected crypts at 7 dpc. Blue arrows indicate clusters of Ki67-stained nuclei at the base of 14 dpc crypts. Scale bar: 50μm. **B)** Estimation of KI67^+^ nuclei in uninfected and *L*. *intracellularis* infected crypts at 3, 7, 14 and 28 dpc. Mean values ± standard error are shown. Y-axis represents the estimated number of Ki67-positive nuclei per crypt.

IF detection of Ki67 protein was used to assess crypt cell proliferation. Ki67^+^ nuclei were observed as discrete foci that were more abundant in the lower halves of uninfected and infected crypts at 3 dpc (Fig 3A). Clusters of Ki67^+^ nuclei were observed in the mid-regions of crypts at 7 dpc (red arrows, Fig 3A). At 14 dpc, Ki67+ nuclei clusters were observed at the bottom of crypts (magenta arrows, Fig 3A). The number of Ki67^+^ clusters increased significantly at 7 dpc (p<0.01) when compared to uninfected crypts and were continuously present along the length of crypts at 14 dpc (p< 0.0001) (Fig 3B).

**Fig 4:**
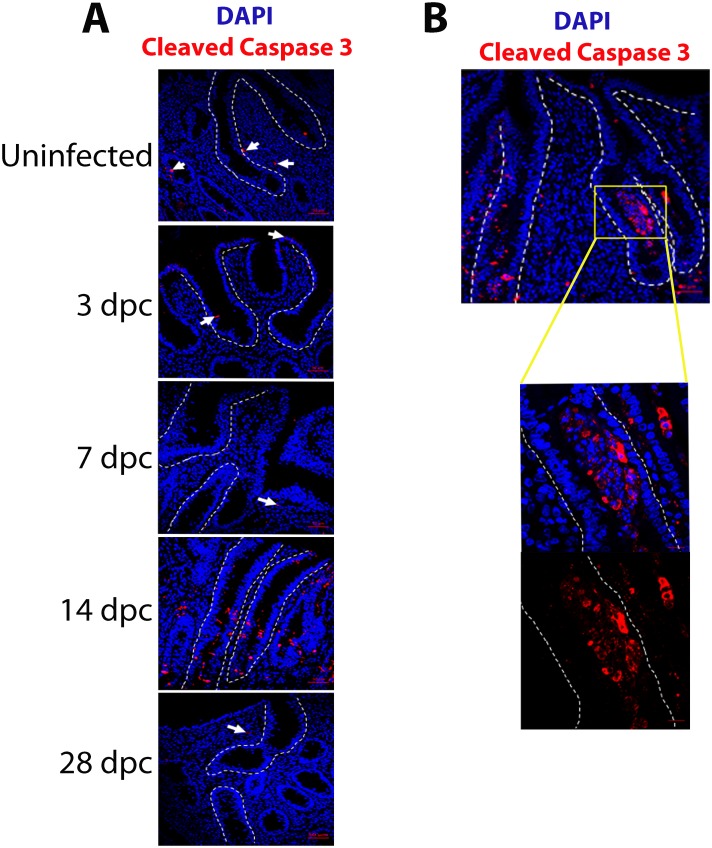
Increase in cleaved Caspase-3 staining in *L*. *intracellularis* infected ileum crypts at 14 dpc. **A)** IF detection of cleaved Caspase-3 in uninfected and *L*. *intracellularis* infected pigs’ ileums of 3, 7, 14 and 28 dpc using confocal microscopy. Anti-cleaved Caspase-3 antibody was detected using Alexa 647-conjugated secondary antibody (red) and DAPI (blue) was used to counterstain the nuclei. Crypts are outlined in white dashed lines. White arrows indicate cleaved Caspase-3 signals observed at the lamina propria and the crypts of uninfected and *L*. *intracellularis* infected ileums at 3, 7 and 28 dpc. Scale bar: 50 μM. **B)** Left: 20x magnification of *L*. *intracellularis* infected pig ileum at 14 dpc; Right: 63x magnification of the crypt region indicated in the white square inset. The crypt is outlined by white dashed lines. Blue: DAPI; Red: cleaved Caspase-3.

IF detection of cleaved Caspase-3 expression was used to assess cell apoptosis in uninfected and infected ileums at 3, 7, 14 and 28 dpc. Staining in uninfected and infected ileums of 3, 7 and 28 dpc was very infrequent to rare, though clearly present (Fig 4A). Most of the cleaved Caspase-3 signal was observed in the surrounding lamina propria, at the tips of the crypts and, rarely, observed in cells lining the crypt (Fig 4A). In crypts at 14 dpc, cleaved Caspase-3 signal was intense and exclusively restricted to the lumens (Fig 4A and 4B).

### Alterations to β-catenin/Wnt signalling during *L*. *intracellularis* infection

**Fig 5:**
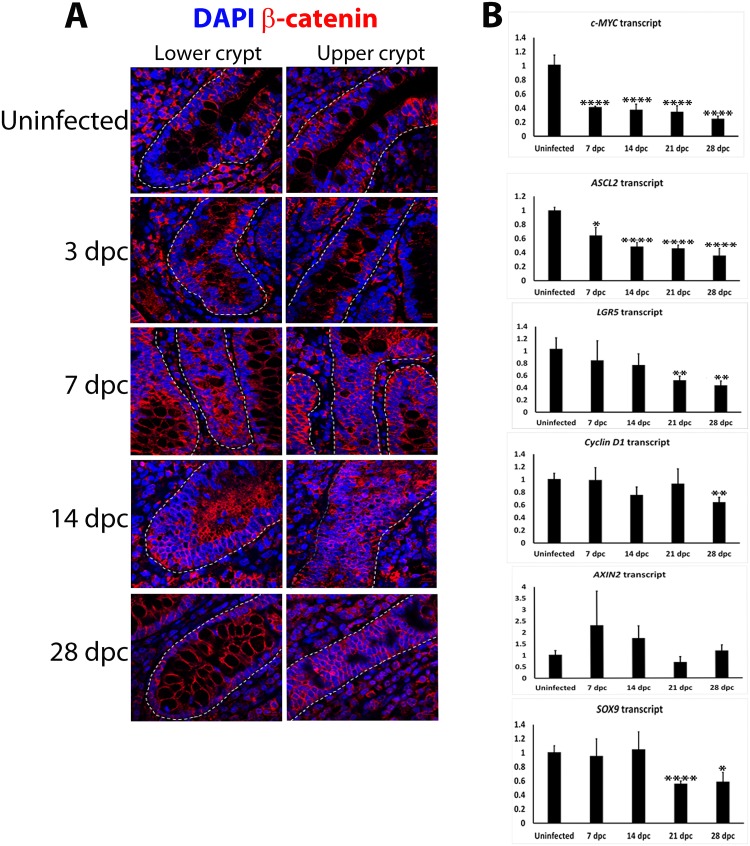
Alterations to β-catenin/Wnt signalling in *L*. *intracellularis* infected ileum crypts at 7 and 14 dpc. **A)** IF detection of β-catenin in uninfected and *L*. *intracelllularis* infected crypts at 3, 7, 14 and 28 dpc using confocal microscopy. The anti-β-catenin antibody was detected using Alexa 647-conjugated secondary antibody (red) while nuclei were counterstained with DAPI (blue). *Left panels*: Lower half of a longitudinal crypt; *Right panels*: Upper half of a longitudinal crypt. Blue: DAPI; Red: β-catenin. Images were acquired at 63x magnification. Scale Bar: 10 μM. **B)** mRNA transcript levels of β-catenin/Wnt target genes in uninfected and *L*. *intracellularis* infected pig ileums at 7, 14, 21 and 28 dpc. Mean values ± standard errors are shown. All RTqPCR results were normalised to GAPDH housekeeping transcript and to uninfected samples [8]. Y-axis of each graph represents normalised transcript levels. *c-MYC* transcript levels. *ASCL2* transcript levels. *LGR5* transcript levels. *Cyclin D1* transcript levels. *AXIN2* transcript levels. *SOX9* transcript levels.

To assess whether the enhanced crypt cell proliferation at 14 dpc was associated with induced β-catenin/Wnt signalling, IF detection of β-catenin was performed. β-catenin was localised to the cell membrane and cytoplasm of uninfected crypts and infected crypts at 3 and 28 dpc. Nuclear β-catenin staining and a pronounced membranous pattern were observed at the base of crypts at 28 dpc (Fig 5A). β-catenin staining was observed mainly in cytosol and cytoplasmic membrane of crypts at 7 and 14 dpc, particularly in the luminal cytosol when compared to uninfected crypts (Fig 5A). β-catenin/Wnt signalling level was further assessed by measuring the transcript levels of its target genes using RTqPCR including *AXIN2*, *c-MYC*, *Cyclin D1* and other responsive genes which specifically mark ISCs, specifically *LGR5*, *ASCL2* and *SOX9* [17, 28–29].

*c-MYC* and *ASCL2* were significantly down-regulated from 7 dpc onwards while *LGR5* was significantly down-regulated from 21 dpc onwards (Fig 5B, 5C and 5D). There was no significant change to *Cyclin D1* level throughout the infection, except at 28 dpc, when it was down-regulated compared to uninfected ileums (p<0.01) (Fig 5E). Together these findings indicated a possible down-regulation of β-catenin/Wnt signalling. The level of down-regulation of AXIN2 and SOX9 was not statistically significant, however (Fig 5F and 5G), perhaps as a result of the difference in bacterial load between biological repeats as depicted in S1 Fig.

**Fig 6:**
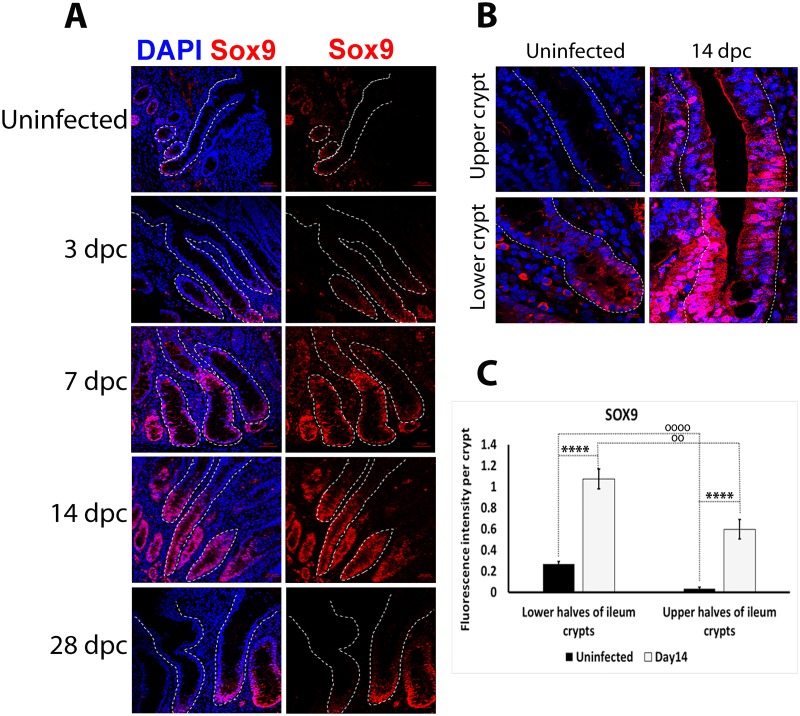
Enhanced SOX9 staining intensity in *L*. *intracellularis* infected ileum crypts at 7 and 14 dpc. **A)** IF detection of SOX9 in uninfected and *L*. *intracellularis* infected crypts at 3, 7, 14 and 28 dpc using confocal microscopy. Anti-SOX9 antibody detected using Alexa 647-conjugated secondary antibody (red). Nuclei were counterstained by DAPI (blue). *Left panels*: Merged DAPI (blue) and SOX9 (red) channels; *Right panels*: SOX9 channel (red). Crypts are outlined with white dashed lines. Scale bar: 50μm. **B)** Comparison of SOX9 (in red) staining in uninfected and *L*. *intracellularis* infected crypts at 14 dpc. Nuclei were counterstained with DAPI (in blue). *Top panels*: Upper halves of crypts; *Bottom Panels*: Lower halves of crypts; *Left panels*: Uninfected crypts; *Right panels*: 14 dpc crypts. Crypts are outlined in white dashed lines. Scale bars: 10 μm. **C)** Quantification of SOX9 fluorescence signal intensity in uninfected and *L*. *intracellularis* infected crypts of pigs at 14 dpc using ImageJ 1.49S. Mean value ± Standard error are shown. Y-axis represents fluorescence intensity of SOX9 antigen staining per crypt. Stars (****) denotes p<0.0001 for comparison of SOX9 staining intensity between uninfected and *L*. *intracellularis* infected crypts at 14 dpc. Circles (o) represent p-values for comparison of SOX9 staining intensity between lower and upper halves of 14 dpc (oo, p<0.01) and uninfected (oooo, p<0.0001) crypts.

SOX9 expression was measured using IF during the course of the infection. In uninfected and infected crypts at 3 and 28 dpc, SOX9 signal was restricted to the crypt base, with obvious nuclear localisation or strong nuclear affinity, and a reduction in signal intensity ascending the crypt (Fig 6A, 6B and 6C). The intensity of nuclear and cytoplasmic SOX9 staining increased along the length of crypts at 7 and 14 dpc compared to uninfected crypts (Fig 6A, 6B and 6C). While overall SOX9 staining was significantly stronger in crypts at 14 dpc compared to uninfected crypts (Fig 6B and 6C showing p-values), SOX9 signal was of significantly lower intensity (p<0.001) in the upper halves of the crypts compare to the base (Fig 6B and 6C).

Taken together these results suggest a differential regulation of β-catenin/Wnt target genes associated with alterations to β-catenin/Wnt signalling during the course of *L*. *intracellularis* infection.

### Induction of Notch signalling during *L*. *intracellularis* infection

**Fig 7:**
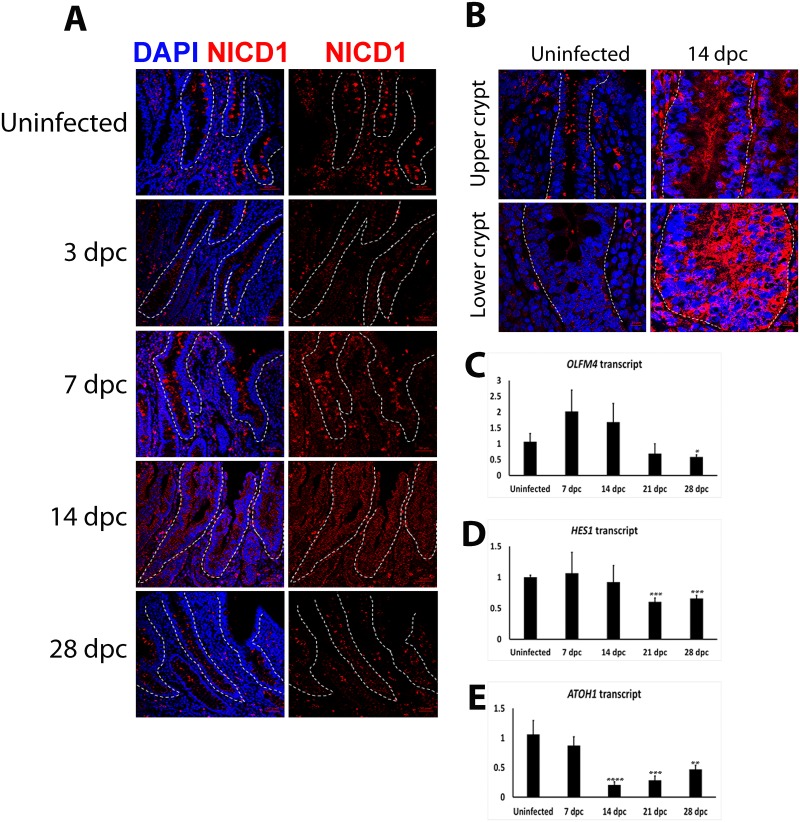
Induction of Notch signalling pathway in *L*. *intracellularis* infected ileum crypts at 14 dpc. **A)** IF detection of Notch-1 receptor intracellular domain/C-terminus (NICD1) in uninfected and *L*. *intracellularis* infected crypts at 3, 7, 14 and 28 dpc using confocal microscopy. Anti-NICD1 antibody was detected using Alexa 647-conjugated secondary antibody (red). Nuclei were counterstained using DAPI (blue). *Left panels*: Merged NICD1 (red) and DAPI (blue) channels; *Right panels*: NICD1 channel (red). Crypts are outlined in white dash lines. Scale bar: 50μm. **B)** Comparison of NICD1 staining (in red) in uninfected and *L*. *intracellularis* infected crypts at 14 dpc. Nuclei were counterstained in DAPI (blue). *Top panels*: Upper halves of crypts; *Bottom Panels*: Lower halves of crypts; *Left panels*: Uninfected crypts; *Right panels*: 14 dpc crypts. Crypts were outlined in white dashed lines. Scale bar: 10μm. **C–E)** mRNA transcript levels of *OLFM4*, *HES1* and *ATOH1* in uninfected and *L*. *intracellularis* infected pig’ ileums at 7, 14, 21 and 28 dpc. Mean values ± Standard error are shown. All RTqPCR results were normalised to GAPDH housekeeping transcript and to uninfected samples [8]. Y-axis of each graphs represents the respective normalised transcript level. **D)** *OLFM4* transcript levels. **E)** *HES1* transcript levels. **F)** *ATOH1* transcript levels.

To assess whether the depletion of MUC2 at 14 dpc was caused by the induction of Notch-1 signalling, the expression of the intracellular domain of Notch-1 receptor (NICD1) was examined using IF. Discrete bright cytoplasmic foci were observed along uninfected crypts (Fig 7A) whereas expression was less pronounced in infected crypts at 3 and 28 dpc crypts, characterised by a dimmer NICD1 expression signal. The NICD1 signal was more continuous along infected crypts at 7 and 14 dpc (Fig 7A, 7B and 7C). Taken together these observations suggest that *L*. *intracellularis* infected crypt cells express more Notch-1 receptor and/or have greater Notch signalling at 14 dpc compared to uninfected crypts.

To verify the activation status of Notch signalling, transcript levels of its direct targets, *HES1*, *OLFM4* and *ATOH1* were also assessed [18–24]. Overall *OLFM4* and *HES1* transcript levels were not significantly altered at 7 and 14 dpc (Fig 7D and 7E). However, transcript levels for *OLFM4* (28 dpc) and *HES1* (21 dpc, 28 dpc) were significantly lower than in uninfected crypts. (Fig 7D and 7E). In contrast, *ATOH1* transcript levels were significantly down-regulated at 14 dpc (p<0.0001) compared to uninfected ileums (Fig 7F), and the trend of *ATOH1* expression during the course of *L*. *intracellularis* infection was similar to that of *MUC2* (Figs 2C and 7F).

## Discussion

As an obligate intracellular pathogen, the interaction between *L*. *intracellularis* and its host cells, the immature crypt cells, is crucial in establishing infection. The hallmark lesion of *L*. *intracellularis* infection is thickening of the intestinal mucosa due to crypt enterocyte proliferation [1–3, 5–8]. The underlying molecular reasons for this proliferation are still unknown but it is likely that *L*. *intracellularis* has developed a specific tropism for these crypt lining epithelial cells [1–3, 6–8]. The naturally high turnover of these cells, together with their vertically-oriented migration and differentiation through the crypt-villus axis, most likely enhances multiplication and local spread of the bacteria [8, 11–12, 14]. We and others have shown that the infection of porcine enterocytes with *L*. *intracellularis* is not only associated with crypt epithelial cell proliferation but also down-regulation of specific host mechanisms involved in cell transport and maintenance of mucosal integrity, as well as enhanced inflammation [8–10].

A well-recognised feature of PE is goblet cell depletion [4, 8]. Consistent with previous reports we have confirmed the specific depletion of goblet cell-derived MUC2 at the peak of infection [4, 8]. Secreted MUC2 represents one of the major components of the mucin layer that entraps bacterial flora, hampering their attachment to the epithelial surface [30–32]. The importance of this mucous layer was elegantly demonstrated using *MUC2* knockout mice that developed spontaneous colitis due to increased susceptibility to enteric infections such as *Citrobacter rodentium* [30]. Moreover, *MUC2* mice showed increased permeability in the colonic mucosal layer that leads to enhanced bacterial adhesion to the epithelial cells and the subsequent increased risk of colonic infection [31]. Therefore, we speculate that depletion of goblet cells and mucin-2 may represent, at least in part, a potential “*modus operandi*” of *L*. *intracellularis* infection that could also have significant consequences for the establishment of secondary infections (i.e. dysbiosis), that may in turn, enhance the severity of clinical signs due to PE [32–33]. In addition to altered secretion of mucin 2, the expression of *RELM-B* (resistin-like beta), *TFF2* and *TFF3* (trefoil factors 2 and 3), genes that encode for proteins exclusively produced by goblet cells, was also concomitantly down-regulated at 14 dpc, indicating a loss of mature goblet cells at the peak of infection [8]. In stark contrast to the infection study of *M*. *avium paratuberculosis* a pathogen which specifically invades human small intestinal goblet cells, our preliminary data suggest a lack of co-localisation between *L*. *intracellularis* bacteria antigen and the goblet cell marker, MUC2 and mucin-containing vacuoles (S2A and S2B Fig) [34]. This is consistent with observations from a previous study which, suggests that the loss of goblet cells may not be caused by a direct invasion of *L*. *intracellularis* into this particular cell type, but could be due to inhibition of goblet cells maturation [4]. However, since this observation is based on the staining of parallel sections and the lack of co-staining experiments, future gene expression profiling of *L*. *intracellularis* infected ileum cells could be pursued to further verify the bacterium’s tropism.

In this study we observed a significant increase in cleaved-caspase-3 expression in pro-apoptotic bodies, at the peak of infection, mainly localized in the crypt lumens rather than within the hyperplastic crypt lining cells. We conclude that the lumen–associated apoptosis may be related to the clearance of *L*. *intracellularis*-infected cells by the immune system, an interpretation which is largely consistent with the infiltration of CD163-positive macrophages and neutrophils in that location observed in previous studies [7–9]. This pattern of immune cell infiltration has been previously reported, not just in experimental cases of PE, but also with a variety of intestinal pathogens including *S*. *typhimurium*, *S*. *flexneri*, enteropathogenic *E*. *coli*, human immunodeficiency virus type 1, *Helicobacter*. *pylori*, and *Cryptosporidium*. *parvum* [8,35–36]. However, in contrast with these intestinal infections, the relative paucity of apoptotic cells along the proliferating infected crypt cells was quite striking. This observation suggests that infected crypt cells are either rapidly cleared or that apoptosis of proliferating crypt cells is subdued during PE. Vannucci et al (2012) showed previously that *L*. *intracellularis*-infected crypt cells expressed higher levels of anti-apoptotic transcripts compared to uninfected controls, perhaps providing support for the hypothesis of altered or inhibited apoptosis. The role of apoptosis regulation still merits further research, particular in the context of *L*. *intracellularis* survival in proliferating crypt cells.

The identification of host factors associated with proliferation of crypt cells in PE has been relatively scarce. For example, despite using a genome-wide transcriptional approach to assess experimentally infected pig intestine, we only identified a single gene (CDK1) directly involved in cell cycle regulation with a change in gene expression [8]. To broaden the scope of our investigations into the mechanism of cell proliferation during PE, we opted for a more targeted approach by monitoring the pattern of regulation of factors that have an established function in cell proliferation and/or differentiation of intestinal stem cells. In the present study, we found concomitant down-regulation of *c-MYC* and *ASCL2* during *L*. *intracellularis* infection. *c-MYC* encodes a “master regulator” oncogene involved in the control of many aspects of cell growth and metabolism, while *ASCL2* encodes an ISC-specific marker required for proliferation [28, 37–39]. These results were surprising in light of previous studies suggesting a regulatory role for these factors in ISC proliferation but, nonetheless, they signaled the possibility of an alternative explanation, i.e. *L*. *intracellularis* may orchestrate the proliferation of a subset of crypt cells such as TA and /or absorptive progenitors rather than ISCs. This is supported by studies suggesting that c-MYC might be dispensable in intestinal homeostasis while N-MYC appears to be enriched in the proliferating progenitor cell compartment of murine intestines [40–41].

Previous studies have shown that β-catenin/Wnt signalling exhibits a dose-dependent effect, since forced induction of β-catenin expression in murine colons promotes the expression of genes that confer an expansion of the LGR5^+^ ISC population and reduced progenitor cell proliferation, while lower levels of β-catenin/Wnt signalling enhance proliferation of progenitor cells, but not ISCs [42–43]. In the present study, low levels of β-catenin/Wnt signalling at the peak of infection may have been the result of the enhanced expression of *AXIN2* and *SOX9*, observed in crypts at 7 and 14 dpc (Fig 5F and 5G). Both AXIN2 and SOX9 are negative regulators of β-catenin/Wnt signalling and, actively promote cytoplasmic sequestration and degradation, respectively, of β-catenin [44–48]. This is also consistent with the increased cytoplasmic β-catenin staining observed in crypts at 7 and 14 dpc (Fig 5A). Moreover, the immunofluorescent staining pattern of NICD1 provides a further mechanism for reduced β-catenin/Wnt signalling, since it was greatest in crypts at 14 dpc. This is supported by enhanced Notch signalling repressing β-catenin/Wnt signalling levels in murine colons and human colorectal cell lines, hence down-regulating the expression of β-catenin/Wnt target genes including *c-MYC* [18, 21]. We have also observed a significant down-regulation in the mRNA levels of the β-catenin/Wnt signalling ligand, *WNT3A*, in crypts at 7 and 14 dpc (S3 Fig). This suggests down-regulation of Wnt signalling since WNT3A is also a direct target gene of the Wnt signalling pathway [49–50]. Further characterization of nuclear/cytoplasmic enrichment of β-catenin, its phosphorylation and promoter regulatory activity in *L*. *intracellularis* infected crypts could shed more light on the role of β-catenin/Wnt signalling during the course of PE, since the simultaneous induction of *SOX9* and *AXIN2* expression and Notch signalling in crypts at 7 and 14 dpc often require elevated β-catenin/Wnt signalling levels [51–53]. Finally, further examination of protein and mRNA expression of other Wnt and R-spondin ligands would also be required to dissect the activation state of β-catenin/Wnt signalling due to high functional redundancy among the Wnt ligands [54].

The significant down-regulation of *LGR5* transcript levels after the peak of *L*. *intracellularis* infection suggests that a reduction in the proliferation rate of LGR5-expressing ISCs, or a reduction in LGR5-expressing ISCs cell number, and/or as suggested previously, a significant attenuation of β-catenin/Wnt signalling, may occur at and/or after the height of infection (Fig 5D) [38]. Reduced LGR5-expressing ISCs proliferation was observed in murine colonic crypt hyperplasia caused by *S*. *typhimurium* infection, as well as during the early stages of crypt regeneration after radiation-induced crypt damage [41, 55–57]. The reduction of ISCs proliferative capacity is known to enhance its survival in damaged crypts as observed in previous studies [58, 59]. One way of doing so is to attenuate the pro-proliferative β-catenin/Wnt signalling in small intestinal crypts (*in-vivo* and *in-vitro*), which promotes the survival of ISCs and reduces their apoptotic cell death after radiation-induced damage [59]. The link between the modulation of ISC proliferation capacity and the attenuation of β-catenin/Wnt signalling may be provided by enhanced SOX9 expression in damaged crypts. SOX9 transcription factor functions in a dose-dependent manner, with high SOX9 expression acting to limit the proliferative capacity of ISCs under homeostatic conditions and immediately after radiation-induced damage [41, 44–45, 47, 56]. In this regard, SOX9 has been shown to repress β-catenin/Wnt signalling and the down-regulation of its pro-proliferative target genes such as c-MYC and CCND1 [44–45]. More recently, SOX9 was shown to repress the pro-proliferative insulin signalling pathway by transcriptionally suppressing the expression of its ligand, insulin-like growth factor (IGF)-binding protein 4 [60]. Taken together, enhanced SOX9 expression promotes survival of ISCs in damaged crypts by controlling their proliferation capacity through the suppression of pro-proliferative pathways including β-catenin/Wnt signalling and other signalling networks [41, 44–45, 47, 56, 60]. We have observed significant enhancement of SOX9 expression at the base and along 14 dpc crypts, as well as the up-regulation of SOX9 expression at 14 dpc (Fig 6B and 6C). These suggest responses to *L*. *intracellularis* infection could include a reprogramming of ISCs gene expression such as the up-regulation of the expression of the pro-survival SOX9 gene. Moreover, *OLFM4* transcript, which encodes for a robust ISC marker associated with anti-apoptotic effects, was also highly expressed in 7–14 dpc ileums, further supporting the notion of genetic reprogramming of ISCs to enhance their survival during the early stages of crypt regeneration after damage (Fig 7D) [61]. Further lineage tracing experiments together with the single molecular RNA fluorescent *in situ* hybridization (smFISH) technique to label different cell types along infected crypts, would be needed to investigate changes to ISCs and other crypt cell populations during *L*. *intracellularis* infection.

Low SOX9 expression in the context of active Notch signalling confers a gene expression profile typical of actively dividing TA progenitor cells [18, 56]. In our study, the significant increase in expression of Ki67, SOX9 and NICD1 in crypts at 14 dpc, and the differential expression of SOX9 between upper and lower crypts at 14 dpc (Figs 3A, 3B, 6A, 6C, 7A and 7C) suggested the expansion of either TA and/or an absorptive progenitor cell population at the peak of *L*. *intracellularis* infection. This may occur in a similar way to that observed in *S*. *typhimurium* induced murine colonic crypt hyperplasia [57]. The authors’ (Rodriguez *et al*.,) interpretation was based on the enhanced *SOX9* and *β-CATENIN* transcript levels and PCNA (proliferating TA cells marker) staining intensity, as well as the lack of changes to LGR5 transcript levels, which is similar to our observation in *L*. *intracellularis* infected ileums [57]. Moreover, enhanced SOX9 expression has been highly associated with gastric metaplastic changes caused by *H*. *pylori*, suggesting the importance of SOX9 expression in *H*. *pylori*-induced gastric carcinogenesis [62].

In the present study we have shown a concomitant induction of NICD1 levels and down-regulation of *ATOH1* and *MUC2* at the peak of *L*. *intracellularis* infection. This observation is consistent with active Notch signalling suppression of *ATOH1* and the inhibition of secretory lineage commitment during infection [18–24]. The expression of absorptive enterocyte-specific solute carriers are found to be down-regulated by *L*. *intracellularis* infection [8, 10], indicating the possibility that *L*. *intracellularis* infection also impedes the maturation of absorptive enterocytes. As described previously in mice, Notch signalling appears to be dispensable for absorptive enterocyte maturation after lineage specification [22]. Thus, this prompts future research into identifying the cellular factors promoting absorptive enterocytes maturation that are perturbed by *L*. *intracellularis* infection. We have also observed that ileum infected with the greatest bacterial load manifested the greatest changes in activity in the β-catenin/Wnt and Notch signalling pathways, including expression of downstream target genes, indicating a potential link between infection burden and the kinetics of changes in downstream signalling (S1 Fig). Future studies using enteroids as models to investigate *L*. *intracellularis* infection and pathogenesis could potentially provide insights into the correlation between bacterial load and the kinetics of changes in β-catenin/Wnt and Notch pathways.

Crypt hyperplasia has been observed in other enteric infections, most notably during *C*. *rodentium* and *S*. *typhimurium* infections of mice [57, 63–67]. Thickening and lengthening of *C*. *rodentium* and *S*. *typhimurium* infected crypts have been attributed to extensive crypt cell proliferation similar to that observed in PE [57, 63–67]. Induced crypt cell proliferation has been hypothesized to be a repair mechanism to replace infected crypt cells [63–64, 66]. In the case of *C*. *rodentium* infection, both β-catenin/Wnt and Notch pathways were induced at the peak of infection [63, 66–67]. However, in contrast to *L*. *intracellularis*, significantly higher expression of c-MYC, RELM-B, LGR5 and the Wnt signalling agonist, R-spondin 2, was observed at the peak of *C*. *rodentium* infection [63, 66–67]. Therefore, even though *C*. *rodentium* and *L*. *intracellularis* induce proliferative crypt lesions that are phenotypically very similar, the means by which they do this are likely to be different [5–6, 64].

This study has allowed us to highlight a potential role for the β-catenin/Wnt and Notch signalling pathways during the infection of crypt cells by *L*. *intracellularis*. Our conclusions are mainly based on the observed association between the expression of specific markers and the burden of *L*. *intracellularis* infection. However, this initial work serves as a basis for further mechanistic investigations perhaps using different experimental models. At a fundamental level, *L*. *intracellularis* may serve as a valuable infection model to study the interface between pathways such as Notch and β-catenin/Wnt and various intestinal cell subtypes, particularly in relation to the differentiation of these subtypes and the reprogramming of ISCs during infection.

## Ethics approval and consent to participate

The experimental samples used in this study were derived from a previous PRRSV study conducted by McIntyre et al., 2003. The study was reviewed and approved by the local Institutional Review Boards of the Royal (Dick) School of Veterinary Studies.

## Supporting information

S1 Table List of primers used for RT-qPCR. (DOCX)

S1 Fig Correlation between *L*. *intracellularis* bacterial load in infected pigs with the alterations to B-catenin/Wnt and Notch signalling target genes expressions. A) Comparison of *L*. *intracellularis* bacterial load (*L*. *intracellularis* isolate LR187/5/83 16S rRNA per ng of genomic DNA) in pigs 325 (7 dpc), 352 (14 dpc) and 353 (14 dpc) to the other pigs euthanized at 7 (324 and 327) and 14 dpc (354 and 355). Data was derived from previous study by Smith et al. (2014) and provided by Dr. Tahar Ait-Ali with the authors’ permission. B) Comparisons of *HES1*, *OLFM4*, *SOX9 and AXIN2* mRNA transcript levels in pigs 325 (7 dpc), 352, 353 (14 dpc) with uninfected pigs and other pigs euthanized at 7 (324 and 327) and 14 dpc (354 and 355). Mean values ± standard deviation are shown. C) Comparison of *MUC2* and *ATOH1* mRNA transcript levels in pigs 325 (7 dpc), 352, 353 (14dpc) with uninfected pigs and other pigs euthanized at 7 and 14 dpc. Mean values ± standard deviation are presented. Note that while *MUC2* and *ATOH1* expression in 325 is similar to that of other pigs euthanized at same time point (7dpc), greater discrepancies in *MUC2* and *ATOH1* expression can be observed between 352 and other pigs euthanized at 14dpc. D) Table showing *AXIN2*, *HES1*, *SOX9* and *OLFM4* mRNA transcript levels in pigs 325 (7dpc), 352 (14dpc) and 353 (14dpc). All RTqPCR results were normalised to GAPDH housekeeping transcript and to uninfected samples thus the mRNAs transcripts levels in uninfected pigs are 1.00 [8]. *p-values* shown were based on comparison of the mRNA transcript level between uninfected pigs and pigs 325, 352 and 353. *P-value* shown for Hes-1 derived from comparison between uninfected pigs and pig 352 and 353.
(DOCX)

S2 Fig Lack of significant co-localisation between LI and MUC2 staining in infected crypts. A) IF detection of L. intracelluaris antigen, LI (using monoclonal VPM53 antibody) and MUC2 in two contiguous sections from L. intracellularis infected crypts at 7 dpc. LI staining and anti-MUC2 were detected using FITC (green) and Alexa-647 (red)-conjugated secondary antibodies, respectively. Nuclei counterstained with DAPI. Insets (blue rectangles) represent the region with with MUC2 signal and mucin-containing vacuoles (black circles). The white dash line represents the outline for the apical sides of the crypt. Scale bar: 10μm. B) IF using VPM53 antibody on L. intracellularis infected crypt at 7 dpc. Green: LI; Blue: DAPI. White dashed lines represent the outline of a crypt. Scale bar: 10μm.
(DOCX)

S3 Fig Messenger RNA transcript level of *WNT3A* in healthy and *L*. *intracellularis* infected pig ileums of 7, 14, 21 and 28dpc. Mean values ± standard errors are shown. All RTqPCR results were normalised to GAPDH housekeeping transcript and to uninfected samples [8].
(DOCX)
