# Supplementary material for: Lawsonia intracellularis exploits β-catenin/Wnt and Notch signalling pathways during infection of intestinal crypt to alter cell homeostasis and promote cell proliferation
Source: PLoS One. 2017 Mar 21;12(3):e0173782. doi: 10.1371/journal.pone.0173782 (PMC5360247; doi:10.1371/journal.pone.0173782)
Supplement: S1 Table — (DOCX) [file pone.0173782.s001.docx]

| **Oligo sequence (5' to 3')** | **Oligo name** | **Accession Number** |
| --- | --- | --- |
| GAACGGGGTACAGAAGCAAA | ATOH1 F | ENSSSCT00000010069 |
| TGGACAGCTTCTTGTCGTTG | ATOH1 R | ENSSSCT00000010069 |
| TCGGACTCTCTGCTCTCCTC | c-MYC F | ENSSSCT00000006548 |
| CCCTCTCGCTTCAGGTCA | c-MYC R | ENSSSCT00000006548 |
| TTCCACGGCACAGTCAA | GAPDH F | ENSSSCG00000002888 |
| GCAGGTCAGGTCCACAA | GAPDH R | ENSSSCG00000002888 |
| GTCAGCAAACCGGCTATTGT | OLFM4 F | ENSSSCT00000010352 |
| TGCCTTGGCCATAGGAAATA | OLFM4 R | ENSSSCT00000010352 |
| GAGCTGCTCGACTTCTCCAG | ASCL2 F | NM_001122991.1 (NCBI) |
| TTCCACATAGCCCTTGGTC | ASCL2 R | NM_001122991.1 (NCBI) |
| CTACCGCCTCTTTGGCAAC | MUC2 F | AK231524.1 (GENBANK) |
| TCGAGTCGTCAGTGACCT | MUC2 R | AK231524.1 (GENBANK) |
| AAGGCGGACATTCTGGAAAT | HES1 F | ENSSSCT00000026743 |
| CCTCGTTCATGCACTCACTG | HES1 R | ENSSSCT00000026743 |
| CTGACGGCCGAGAAGTTGT | CYCLIN D1 F | ENSSSCT00000014070 |
| TTGGAGAGGAAGTGCTCGAT | CYCLIN D1 R | ENSSSCT00000014070 |
| GAGGGAGAAATGCGTGGATA | AXIN2 F | ENSSSCT00000018794 |
| GGTTTCAGCTGCTTGGAGAC | AXIN2 R | ENSSSCT00000018794 |
| GAGTGCCAACACCAGTTCC | WNT3A F | ENSSSCT00000031062 |
| AGTCACAGCGAAGGCAACTC | WNT3A R | ENSSSCT00000031062 |


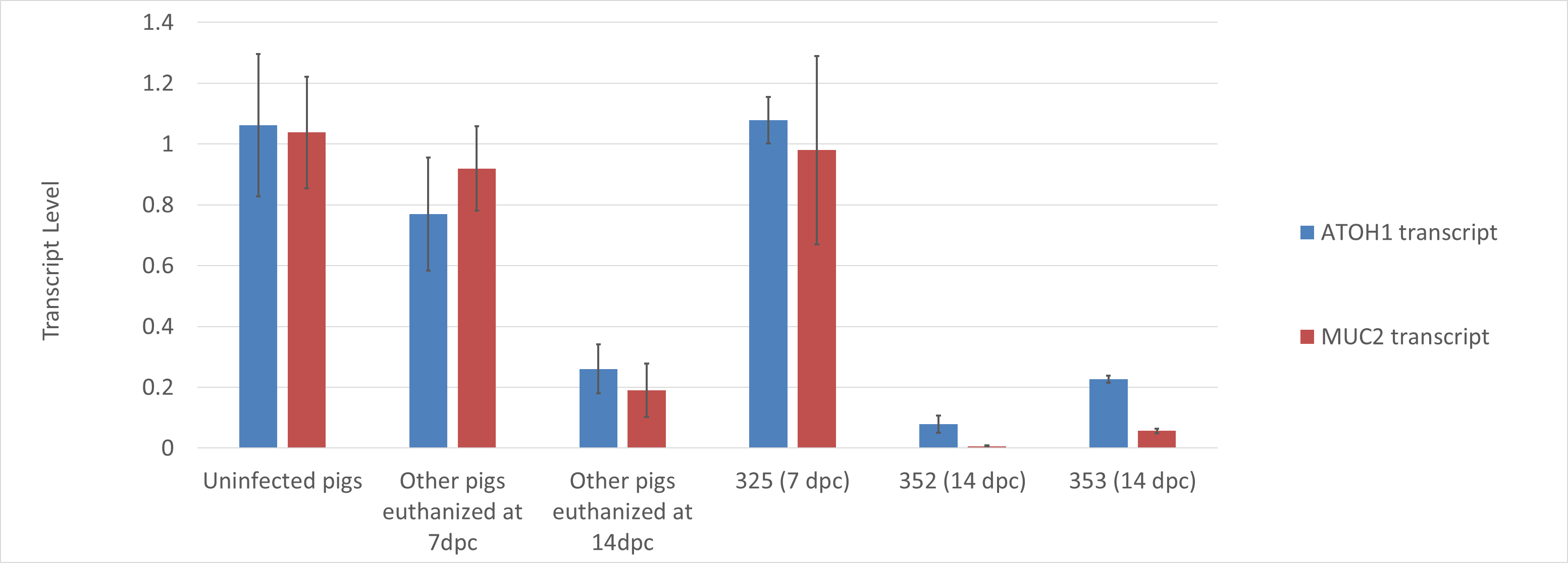


C
