# Supplementary material for: Lawsonia intracellularis exploits β-catenin/Wnt and Notch signalling pathways during infection of intestinal crypt to alter cell homeostasis and promote cell proliferation
Source: PLoS One. 2017 Mar 21;12(3):e0173782. doi: 10.1371/journal.pone.0173782 (PMC5360247; doi:10.1371/journal.pone.0173782)
Supplement: S1 Fig — A) Comparison of L. intracellularis bacterial load (L. intracellularis isolate LR187/5/83 16S rRNA per ng of genomic DNA) in pigs 325 (7 dpc), 352 (14 dpc) and 353 (14 dpc) to the other pigs euthanized at 7 (324 and 327) and 14 dpc (354 and 355). Data was derived from previous study by Smith et al. (2014) and provided by Dr. Tahar Ait-Ali with the authors’ permission. B) Comparisons of HES1, OLFM4, SOX9 and AXIN2 mRNA transcript levels in pigs 325 (7 dpc), 352, 353 (14 dpc) with uninfected pigs and other pigs euthanized at 7 (324 and 327) and 14 dpc (354 and 355). Mean values ± standard deviation are shown. C) Comparison of MUC2 and ATOH1 mRNA transcript levels in pigs 325 (7 dpc), 352, 353 (14dpc) with uninfected pigs and other pigs euthanized at 7 and 14 dpc. Mean values ± standard deviation are presented. Note that while MUC2 and ATOH1 expression in 325 is similar to that of other pigs euthanized at same time point (7dpc), greater discrepancies in MUC2 and ATOH1 expression can be observed between 352 and other pigs euthanized at 14dpc. D) Table showing AXIN2, HES1, SOX9 and OLFM4 mRNA transcript levels in pigs 325 (7dpc), 352 (14dpc) and 353 (14dpc). All RTqPCR results were normalised to GAPDH housekeeping transcript and to uninfected samples thus the mRNAs transcripts levels in uninfected pigs are 1.00 [8]. p-values shown were based on comparison of the mRNA transcript level between uninfected pigs and pigs 325, 352 and 353. P-value shown for Hes-1 derived from comparison between uninfected pigs and pig 352 and 353. (DOCX) [file pone.0173782.s002.docx]

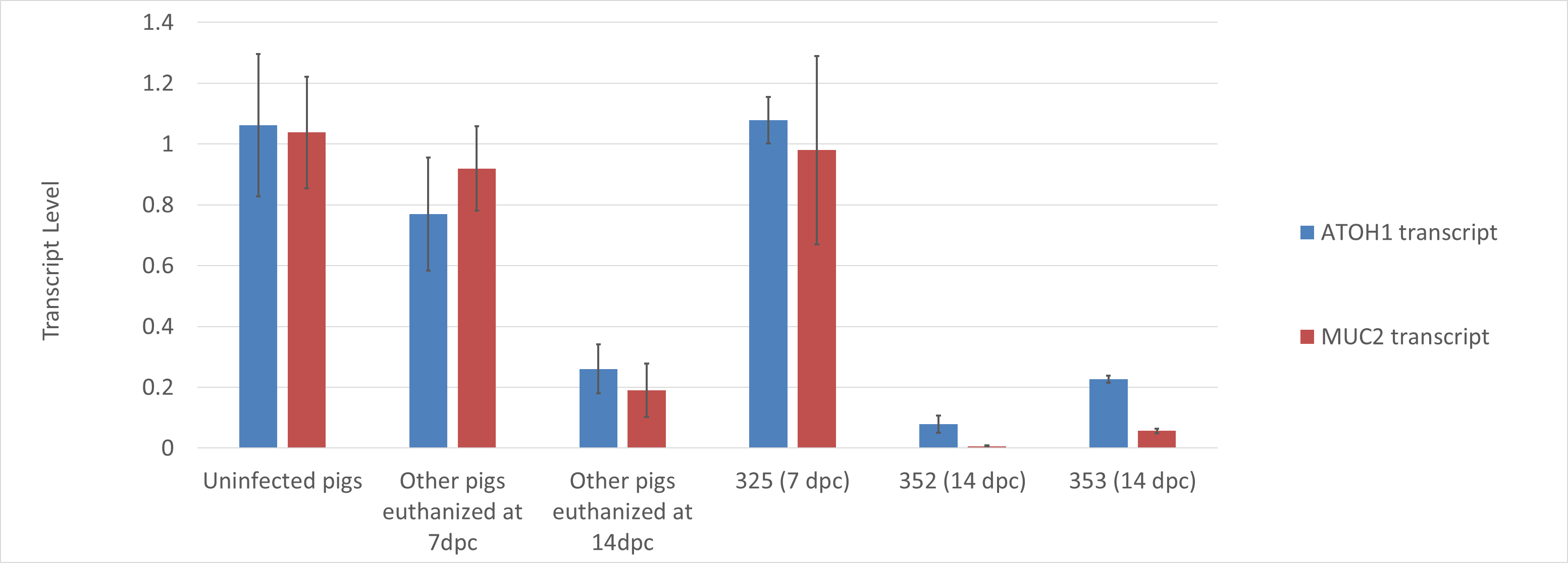


A

C

**
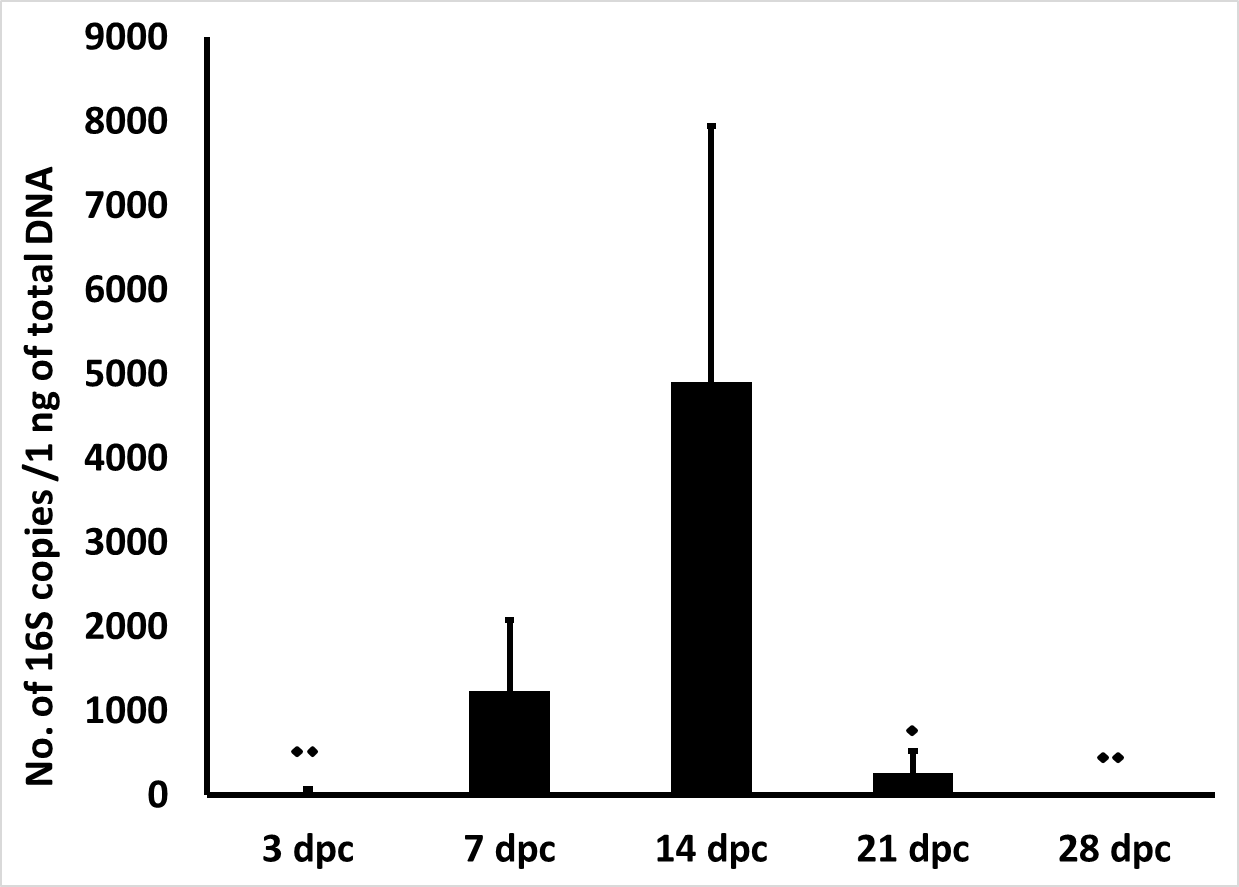
**

B

**
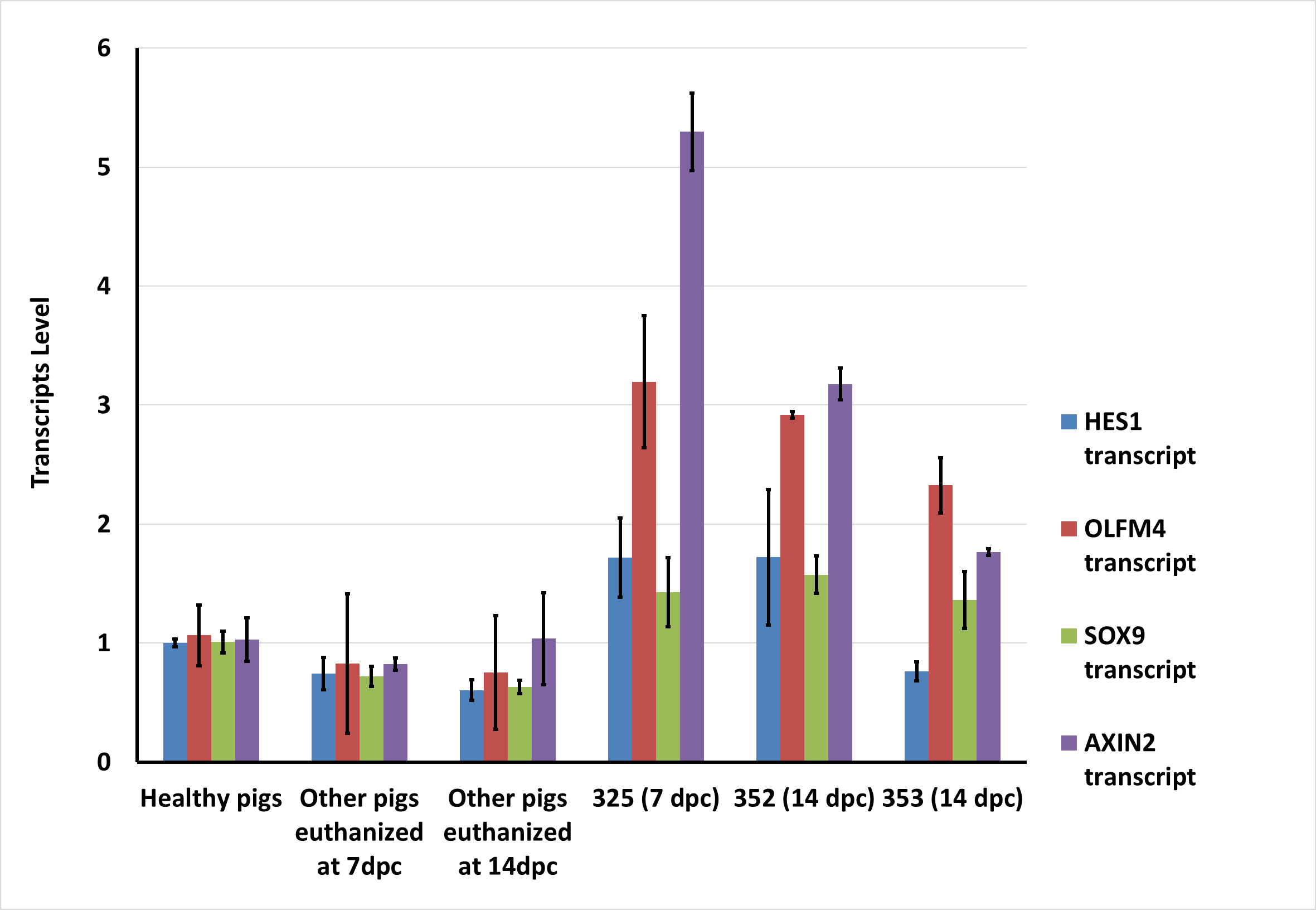
**

C

**
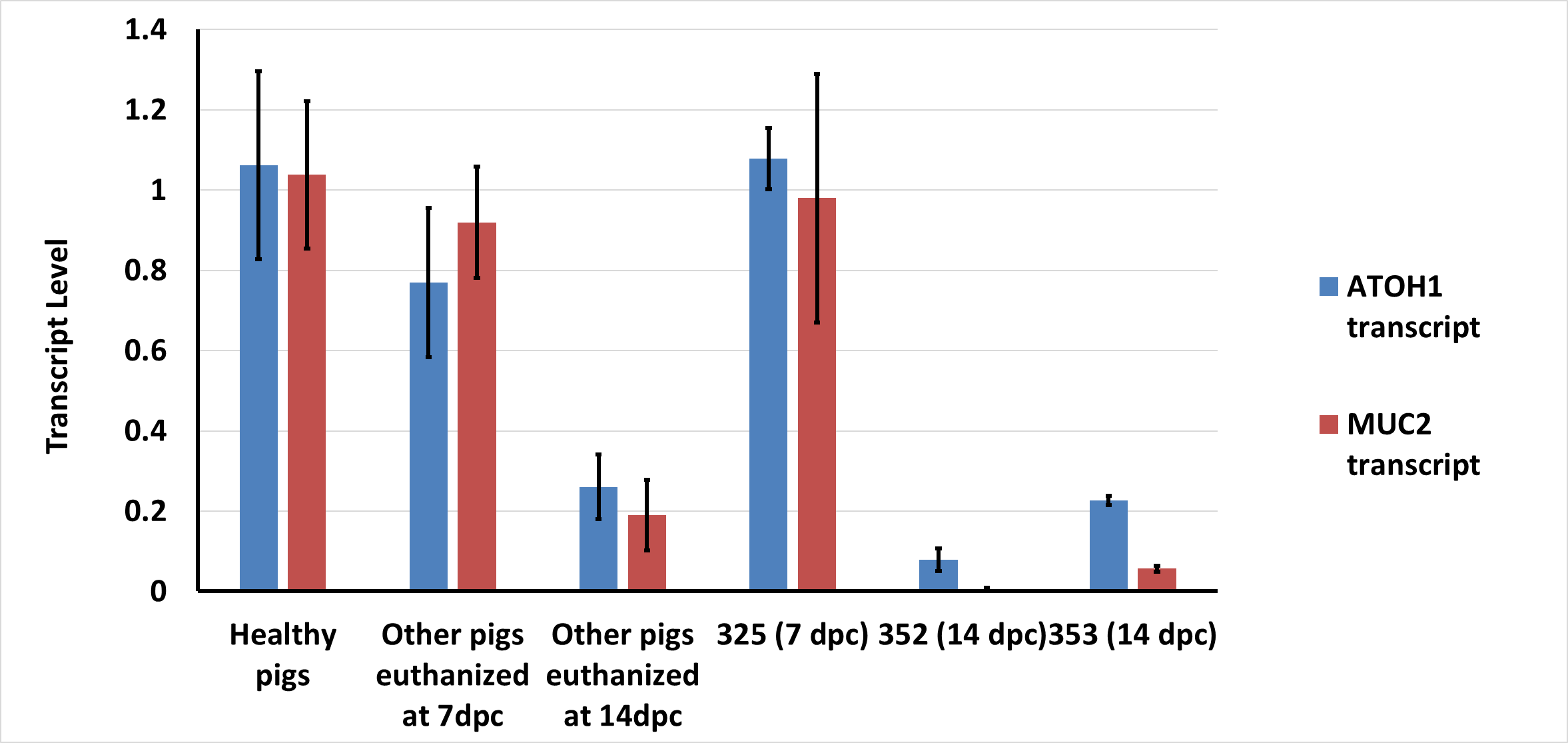
**

**D**

| mRNA transcript | mRNA transcript level | | | |
| --- | --- | --- | --- | --- |
|  | Pig 325 (7dpc) | Pig 352 (14dpc) | Pig 353 (14dpc) | p-values |
| *AXIN2* | 5.29 ±0.08 | 3.17 ±0.06 | 1.77 ±0.02 | p<0.0010 |
| *SOX9* | 1.43 ±0.02 | 1.57 ±0.12 | 1.37 ±0.02 | p<0.0061 |
| *HES1* | 1.72 ±0.21 | 1.71 ±0.25 | 0.76 ±0.030 | P<0.040 |
| *OLFM4* | 3.20 ± 0.20 | 2.92 ±0.05 | 2.32 ±0.15 | p<0.0013 |
