# Supplementary figures and images for: Lawsonia intracellularis exploits β-catenin/Wnt and Notch signalling pathways during infection of intestinal crypt to alter cell homeostasis and promote cell proliferation
Source: PLoS One. 2017 Mar 21;12(3):e0173782. doi: 10.1371/journal.pone.0173782 (PMC5360247; doi:10.1371/journal.pone.0173782)

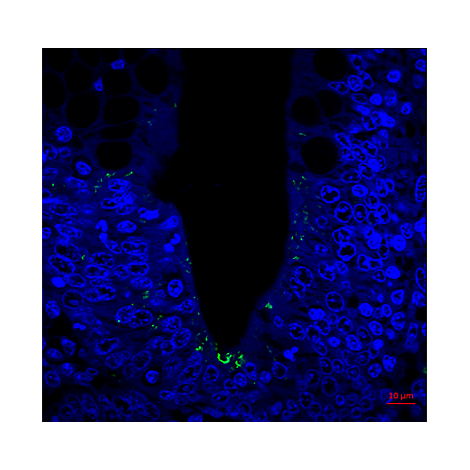

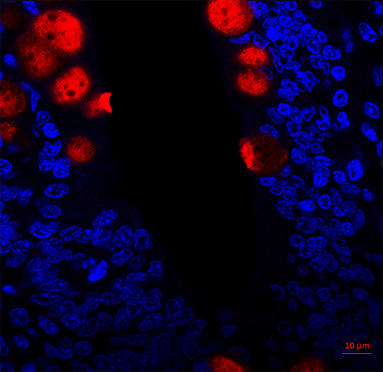

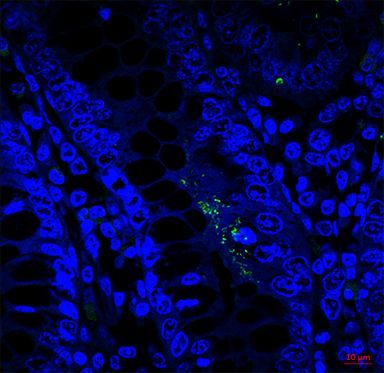


**DAPI MUC2**

**DAPI LI**

A)

B)

**DAPI LI**

Supplement: S2 Fig — A) IF detection of L. intracelluaris antigen, LI (using monoclonal VPM53 antibody) and MUC2 in two contiguous sections from L. intracellularis infected crypts at 7 dpc. LI staining and anti-MUC2 were detected using FITC (green) and Alexa-647 (red)-conjugated secondary antibodies, respectively. Nuclei counterstained with DAPI. Insets (blue rectangles) represent the region with with MUC2 signal and mucin-containing vacuoles (black circles). The white dash line represents the outline for the apical sides of the crypt. Scale bar: 10μm. B) IF using VPM53 antibody on L. intracellularis infected crypt at 7 dpc. Green: LI; Blue: DAPI. White dashed lines represent the outline of a crypt. Scale bar: 10μm. (DOCX) [file pone.0173782.s003.docx]
