# Supplementary material for: Lawsonia intracellularis exploits β-catenin/Wnt and Notch signalling pathways during infection of intestinal crypt to alter cell homeostasis and promote cell proliferation
Source: PLoS One. 2017 Mar 21;12(3):e0173782. doi: 10.1371/journal.pone.0173782 (PMC5360247; doi:10.1371/journal.pone.0173782)
Supplement: Supplementary file 4 [file pone.0173782.s004.docx]

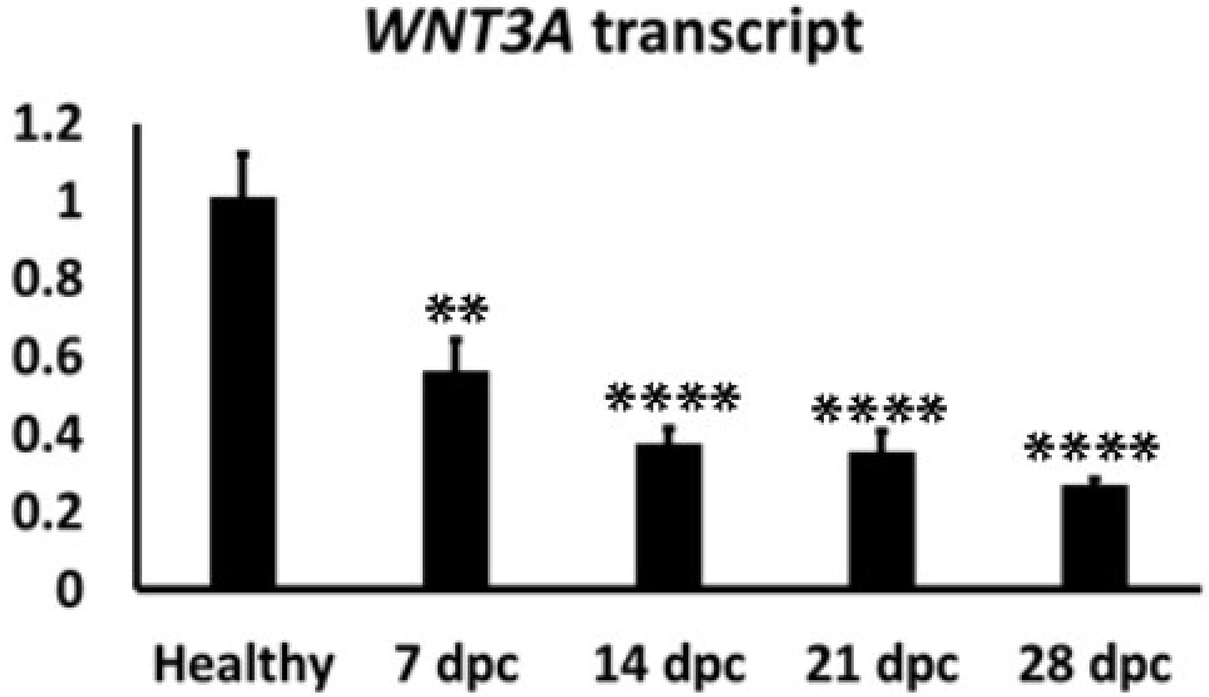
WNT3A transcript
1.2
1
0.8
0.6
0.4
0.2
0
**
****
****
****
Healthy
7 dpc
14 dpc
21 dpc
28 dpc
